# Generalized allergic reaction in response to exercise due to strawberry gibberellin-regulated protein: a case report

**DOI:** 10.1186/s13223-022-00692-0

**Published:** 2022-06-11

**Authors:** Chisato Inuo, Fumiko Okazaki, Rie Shiraki, Yutaka Tanaka, Keiko Momma, Yasuto Kondo, Hiroshi Narita

**Affiliations:** 1 Department of Allergy, Kanagawa Children’s Medical Center, 2-138-4 Mutsukawa, Minami-ku, Yokohama, Kanagawa 232-8555 Japan; 2 Department of Food Science and Human Nutrition Faculty of Agriculture, Ryukoku University, 1-5 Yokotani, Seta Oe-cho, Otsu, Shiga 520-2194 Japan; 3 Department of Food Science, Kyoto Women’s University, 35 Kitahiyoshi-cho, Imakumano, Higashiyama-ku, Kyoto, 605-8501 Japan; 4 Department of Pediatrics, Fujita Health University Bantane Hospital, 3-6-10 Otobashi, Nakagawa-Ku, Nagoya, Aichi Japan; 5 Kyoto College of Nutritional and Medical Sciences, 18 Setogawa-cho, Sagatenryuji, Ukyo-ku, Kyoto, Japan

**Keywords:** Strawberry, Gibberellin-regulated protein, Fruits, Pollen-food allergy syndrome, Peach

## Abstract

**Background:**

The Rosaceae family includes fruits, such as peach, apple, Japanese apricot, cherry (Prunoideae subfamily), and strawberry (Rosoideae subfamily). The allergens responsible for Rosaceae fruit allergies have been reported to include Bet v 1 and profilin, which mainly cause oral symptoms, and lipid transfer protein (LTP). Recently, gibberellin-regulated protein (GRP) has been identified as an allergen that induces generalized symptoms in peach-, orange-, and plum-related allergies. Most patients with food allergies induced by GRP show allergic symptoms accompanied by cofactors, such as exercise or drugs. To date, there are very few reports of generalized symptoms induced by strawberry.

**Case presentation:**

We evaluated the reactivity of strawberry GRP in a 15-year-old boy who was confirmed to have generalized symptoms induced by strawberry with exercise using an oral food challenge test (OFCT). The patient’s serum exhibited a strong positive reaction to strawberry GRP but not to peach GRP or peach LTP. The patient’s basophils reacted to strawberry and peach GRP but not to peach LTP.

**Conclusions:**

Strawberry GRP may be a causative component for strawberry with exercise-induced generalized symptoms in this patient. This is the first study to investigate the role of GRP in strawberry with cofactor-induced allergic symptoms. Further epidemiological and clinical researches are necessary to improve diagnostic and therapeutic approaches for patients with strawberry allergy.

## Background

The Rosaceae family includes fruits, such as peach, apple, Japanese apricot, cherry (Prunoideae subfamily), and strawberry (Rosoideae subfamily). The allergens responsible for Rosaceae fruit allergies have been reported to include Bet v 1 and profilin, which mainly cause oral symptoms, and lipid transfer protein (LTP) and gibberellin-regulated protein (GRP), which cause systemic symptoms [1].

Strawberries are cultivated and eaten worldwide. Cases of strawberry allergy have been reported previously [2–7]. Three allergens (Fra a 1, 3, and 4) have been identified for strawberries [4]. Fra a 1 is Bet v 1 homolog that has already lost the immunoglobulin (Ig) E-binding activities through heat or enzyme treatment. Therefore, a Bet v 1 homolog causes immediate allergy localized in the oral mucosa; it rarely causes generalized symptoms. Fra a 4 belongs to the profilin family, which is the most widespread allergen throughout the plant. Moreover, the allergens of the profilin family are not heat-stable and are vulnerable to gastric digestion. Fra a 3 belongs to the LTP family. Some studies have reported that Pru p 3 (LTP in peach) causes generalized symptoms [1]. Interestingly, a previous report suggested that Fra a 3 is less potent than peach or apple LTP [5]. To date, there are very few reports of generalized symptoms induced by strawberry [2, 6, 7].

GRP has been identified as another allergen that induces generalized symptoms in peach-, orange-, and plum-related allergies [7–14]. Most patients with food allergies induced by GRP show allergic symptoms accompanied by cofactors, such as exercise or drugs [13]. Thus, we evaluated the reactivity of strawberry GRP in a patient who was confirmed to have generalized symptoms induced by strawberry with exercise using an oral food challenge test (OFCT).

## Case presentation

A 15-year-old boy presented with three episodes of allergic symptoms induced by exercise following ingestion of certain fruits. First, he had generalized urticaria induced by exercise after ingesting mixed fruit with canned peach. Moreover, he had generalized urticaria and cough induced by exercise after ingesting yogurt and apple. Furthermore, he had generalized urticaria and swollen lips induced by walking fast to school after ingesting strawberries and yogurt. He had no prior history of allergic symptoms presenting after ingestion of other foods. The patient underwent OFCTs, which included ingesting 10 fresh strawberries, one commercially available yogurt cup, one whole canned peach, or one and a half apples along with aspirin, followed by a 500-m sprint after 15 min, each on a different day. He experienced generalized urticaria after fruit consumption and exercise but not after eating yogurt with aspirin and/or exercise. He was diagnosed with exercise-induced strawberry, peach, and apple allergies. He had pollinosis, and his specific IgE levels (ImmunoCAP; ThermoFisher Scientific, Waltham, MA) for Japanese cedar and cypress pollen were 26.4 and 9.73 U_A_/mL, respectively, whereas those for birch, alder pollen, and rBet v 1 were normal (< 0.30 U_A_/mL). His fruit-specific IgE levels, including those for strawberry, peach, apple, rPru p1, rPru p 3, rPru p 4, rMal d 1, and rMal d 3, were normal (Table 1).

**Table 1:** Clinical backgrounds of the present patient and control patients

		Present patient	Control patient 1	Control patient 2
Clinical symptoms				
Strawberry				
Generalized symptoms		** + **	−	**−**
Localized symptoms		** + **	** + **	** + **
Peach				
Generalized symptoms		** + **	** + **	**−**
Localized symptoms		** + **	** + **	** + **
Apple				
Generalized symptoms		** + **	**−**	**−**
Localized symptoms		** + **	** + **	** + **
sIgE (U_A_/mL)				
Strawberry sIgE	< 0.30		< 0.30	0.76
Peach sIgE	< 0.30		< 0.30	2.13
Apple sIgE	< 0.30		< 0.30	3.82
Japanese Cedar sIgE	26.4		58.1	78.4
Japanese Cypress sIgE	9.73		14.6	11.8
Birch sIgE	< 0.30		< 0.30	50.8
Alder sIgE	< 0.30		< 0.30	38.4

*sIgE* specific immunoglobulin E.

We performed a skin prick test with fresh strawberries using the “prick-prick” procedure. Histamine dihydrochloride (10 mg/mL) and saline were used as positive and negative controls, respectively. The patient exhibited positive reactions for strawberry (wheal, 4 mm) and the positive control (wheal, 10 mm) in the prick test and negative reactions for the negative control (wheal, 0 mm).

**Fig. 1:**
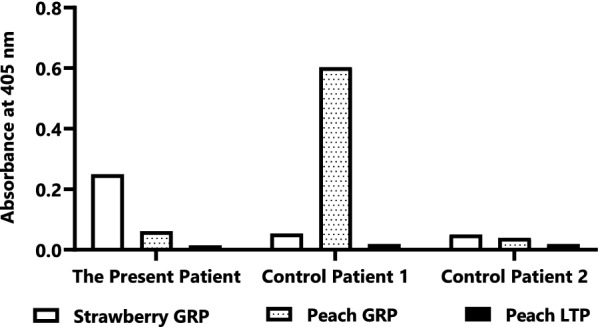
ELISA results for the patient’s serum and the sera of control patients. The patient’s serum showed a strong positive result for strawberry GRP. The serum of Control Patient 1 with severe peach allergy exhibited a strong positive result for peach GRP. The serum of Control Patient 2 with localized symptoms induced by peach and strawberry exhibited a negative result for peach and strawberry GRPs. The sera of all patients exhibited negative results for peach LTP. *ELISA* enzyme-linked immunosorbent assay, *GRP* gibberellin-regulated protein, *LTP* lipid transfer protein

For component analysis, we prepared peach GRP and LTP by immunoaffinity columns using monoclonal antibodies, as reported previously [10]. Strawberry GRP was purified using a monoclonal antibody against peach GRP, which exhibited cross-reactivity to strawberry GRP. Each purified protein showed a single band on sodium dodecyl sulfate–polyacrylamide gel electrophoresis under reducing or non-reducing conditions. The specific IgE levels to each protein were measured by enzyme-linked immunosorbent assay (ELISA) with peroxidase-labeled anti-human IgE (SeraCare Life Sciences, Milford, MA). Data exhibiting absorbance at values 450 nm over the mean + three standard deviations of those of negative controls (four non-allergic individuals) were considered positive. We included control patients with strawberry, peach, and apple allergies among the patients of our hospital to evaluate the immunological difference in the reactivity of systemic and local symptoms to strawberries and peaches. Control Patient 1 had episodic generalized symptoms after ingesting peaches and mild oropharyngeal pruritus after ingesting strawberries without generalized symptoms. Control Patient 2 had mild oropharyngeal pruritus and swelling of the lips after ingesting peaches, apples, and strawberries without presenting generalized symptoms. Differences in the clinical backgrounds and allergen-specific IgEs between the case patient and controls are shown in Table 1. The patient’s serum exhibited a strong positive result to strawberry GRP but a negative result to peach GRP and peach LTP. Control Patient 1 exhibited a strong positive result to peach GRP but not to strawberry GRP or peach LTP. Control Patient 2 did not react to any component. These results are shown in Fig. 1.

**Fig. 2:**
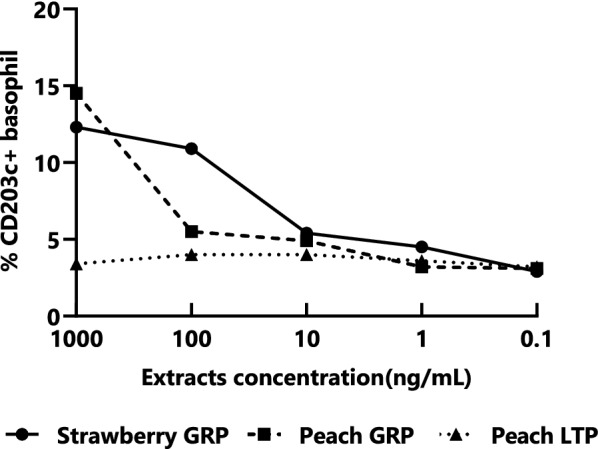
Basophil activation by strawberry GRP, peach GRP, and peach LTP extracts. The basophils of the present patient reacted to strawberry and peach GRPs but not to peach LTP. *GRP* gibberellin-regulated protein, *LTP* lipid transfer protein

Basophil activation (BA) was tested using an allergenicity kit (Beckman Coulter, Fullerton, CA), as previously described [15]. Samples were analyzed using a FACSCalibur™ cell analyzer with CellQuest software (Becton Dickinson, Franklin Lakes, NJ). Basophils were identified based on their forward- and side-scatter properties, absence of CD3, and presence of CRTH2. We evaluated allergen-specific BA using serially diluted components (five concentrations, 0.1–1000 ng/mL of total protein). We constructed dose-response curves for BA based on tenfold decreasing concentrations of the respective extracts. The area under the curve (AUC) of BA was evaluated. The patient’s basophils reacted with strawberry and peach GRP but not with peach LTP (Fig. 2). The AUCs of BA due to strawberry and peach GRP were 11,221 and 9507, respectively. Basophils of Control Patient 2 did not react with these components (highest value: 4.0%). The BA of Control Patient 1 could not be evaluated.

## Discussion and conclusions

### The summary of the patient and the reactivity of strawberry GRP

This patient was confirmed to have strawberry, peach, and apple allergies using OFCTs. The immunological evaluations against strawberry and peach GRP had been performed. The patient’s basophils and serum reacted with strawberry and peach GRP. Those of control patients who showed limited perioral symptoms without generalized symptoms after ingesting strawberries did not react to strawberry GRP.

### The characters of GRP and cofactors

Strawberries are eaten raw and in processed foods, such as jam, yogurt, and cake. Tuppo et al. revealed that peach GRP is heat-stable [8], which suggests that strawberry GRP might be heat-stable and induce allergy symptoms after ingestion of strawberry-containing processed food.

Most patients with sensitization to peach GRP were not sensitized to Rosaceae family pollens, including birch and alder pollen [13]. Additionally, a relationship between cypress pollen allergy and GRP has been reported [14]. Our patient’s specific IgE levels are consistent with those reported previously.

This patient had no symptoms after ingestion of strawberries without exercise. He exhibited no respiratory or cardiological symptoms and had never experienced anaphylaxis. The symptoms induced by fruits required a cofactor, such as exercise and/or aspirin ingestion. Klingebiel et al. showed that BA in patients allergic to peach GRP with clinical reactions requiring cofactors was lower than that without cofactor requirement [14], which is consistent with our patient’s BA. The low number of positive results in OFCTs against strawberries in a previous study may be because no cofactors were added to the OFCTs [6]. Cofactors, such as exercise, are ones that lower the threshold for allergic reactions to food and increase the likelihood of a systemic reaction. Thus, in the present case, the sensitization to GRP was discovered after the symptoms occurred. We hope that strawberry GRP measurement will become more common to prevent allergic symptoms from occurring.

### The reactivity of peach GRP and LTP

The serum of this patient did not react to peach GRP. The reaction of some patients’ sera to peach GRP was much weaker than that of BAs in a previous report [14]. Our previous report showed that some patients’ sera exhibit weaker reactions to native peach GRP than to recombinant peach GRP [10]. The reactivity of peach GRP in ELISA and BA when assessing peach-induced generalized symptoms should be further examined.

We could not extract strawberry LTP; therefore, we could not evaluate the reaction of the present patient’s serum to strawberry LTP. Reactions to apple LTP (Mal d 3) and peach LTP (Pru p 3) in the present patient were negative. A previous study showed that rFra a 3 (a strawberry LTP) is 73−77% homologous to Mal d 3, and all patients who tested negative for Mal d 3 also tested negative for rFra a 3 in that study [5]. It is unlikely that strawberry LTP contributed to the present patient's systemic symptoms.

### Limitations

This study had some limitations. First, tests for allergen components in strawberry are not available commercially and, hence, were not performed in this patient. Second, it remains unclear whether the primary sensitization to strawberry GRP was caused by peach or pollen.

### Conclusion

Strawberry GRP may be a causative component for strawberry with exercise-induced generalized symptoms in this patient. To our knowledge, this is the first study to investigate the role of GRP in strawberry with cofactor-induced allergic symptoms. Further epidemiological and clinical researches are necessary to improve diagnostic and therapeutic approaches for patients with strawberry allergy.

## Data Availability

All data generated or analyzed during this study are included in this article. Further inquiries can be directed to the corresponding author.

## Abbreviations

AUC: Area under the curve
BA: Basophil activation
ELISA: Enzyme-linked immunosorbent assay
GRP: Gibberellin-regulated protein
LTP: Lipid transfer protein
OFCT: Oral food challenge test
